# Hierarchical genetic structure in an evolving species complex: Insights from genome wide ddRAD data in *Sebastes mentella*

**DOI:** 10.1371/journal.pone.0251976

**Published:** 2021-05-27

**Authors:** Atal Saha, Matthew Kent, Lorenz Hauser, Daniel P. Drinan, Einar E. Nielsen, Jon-Ivar Westgaard, Sigbjørn Lien, Torild Johansen

**Affiliations:** 1 Division of Population Genetics, Department of Zoology, Stockholm University, Stockholm, Sweden; 2 Institute of Marine Research, Tromsø Department, Tromsø, Norway; 3 Centre for Integrative Genetics, Department of Animal and Aquacultural Sciences, Faculty of Biosciences, Norwegian University of Life Sciences, Ås, Norway; 4 School of Aquatic and Fishery Sciences, University of Washington, Seattle, Washington, United States of America; 5 DTU Aqua–National Institute of Aquatic Resources, Charlottenlund, Denmark; Universidad de los Andes, COLOMBIA

## Abstract

The diverse biology and ecology of marine organisms may lead to complex patterns of intraspecific diversity for both neutral and adaptive genetic variation. *Sebastes mentella* displays a particular life-history as livebearers, for which existence of multiple ecotypes has been suspected to complicate the genetic population structure of the species. Double digest restriction-site associated DNA was used to investigate genetic population structure in *S*. *mentella* and to scan for evidence of selection. In total, 42,288 SNPs were detected in 277 fish, and 1,943 neutral and 97 tentatively adaptive loci were selected following stringent filtration. Unprecedented levels of genetic differentiation were found among the previously defined ‘shallow pelagic’, ‘deep pelagic’ and ‘demersal slope’ ecotypes, with overall mean *F*_ST_ = 0.05 and 0.24 in neutral and outlier SNPs, respectively. Bayesian computation estimated a concurrent and historical divergence among these three ecotypes and evidence of local adaptation was found in the *S*. *mentella* genome. Overall, these findings imply that the depth-defined habitat divergence of *S*. *mentella* has led to reproductive isolation and possibly adaptive radiation among these ecotypes. Additional sub-structuring was detected within the ‘shallow’ and ‘deep’ pelagic ecotypes. Population assignment of individual fish showed more than 94% agreement between results based on SNP and previously generated microsatellite data, but the SNP data provided a lower estimate of hybridization among the ecotypes than that by microsatellite data. We identified a SNP panel with only 21 loci to discriminate populations in mixed samples based on a machine-learning algorithm. This first SNP based investigation clarifies the population structure of *S*. *mentella*, and provides novel and high-resolution genomic tools for future investigations. The insights and tools provided here can readily be incorporated into the management of *S*. *mentella* and serve as a template for other exploited marine species exhibiting similar complex life history traits.

## Introduction

Disentangling complex genetic structure is needed for conservation of intraspecific biodiversity and future evolutionary potential of exploited species [1]. Evidence of genetically structured populations within many marine species has increased during the last decades [for review see 2, 3]. Patterns of genetic diversity across widely distributed marine taxa can be different depending on associated ecological and biological traits. For example, life history [e.g. 4] and ecological [e.g. 5] traits of a species can be major drivers of biological diversification and their interplay can lead to the formation of a hierarchical genetic population structure, of which the delineation can be of key importance to ensure sustainable management and conservation.

**Fig 1:**
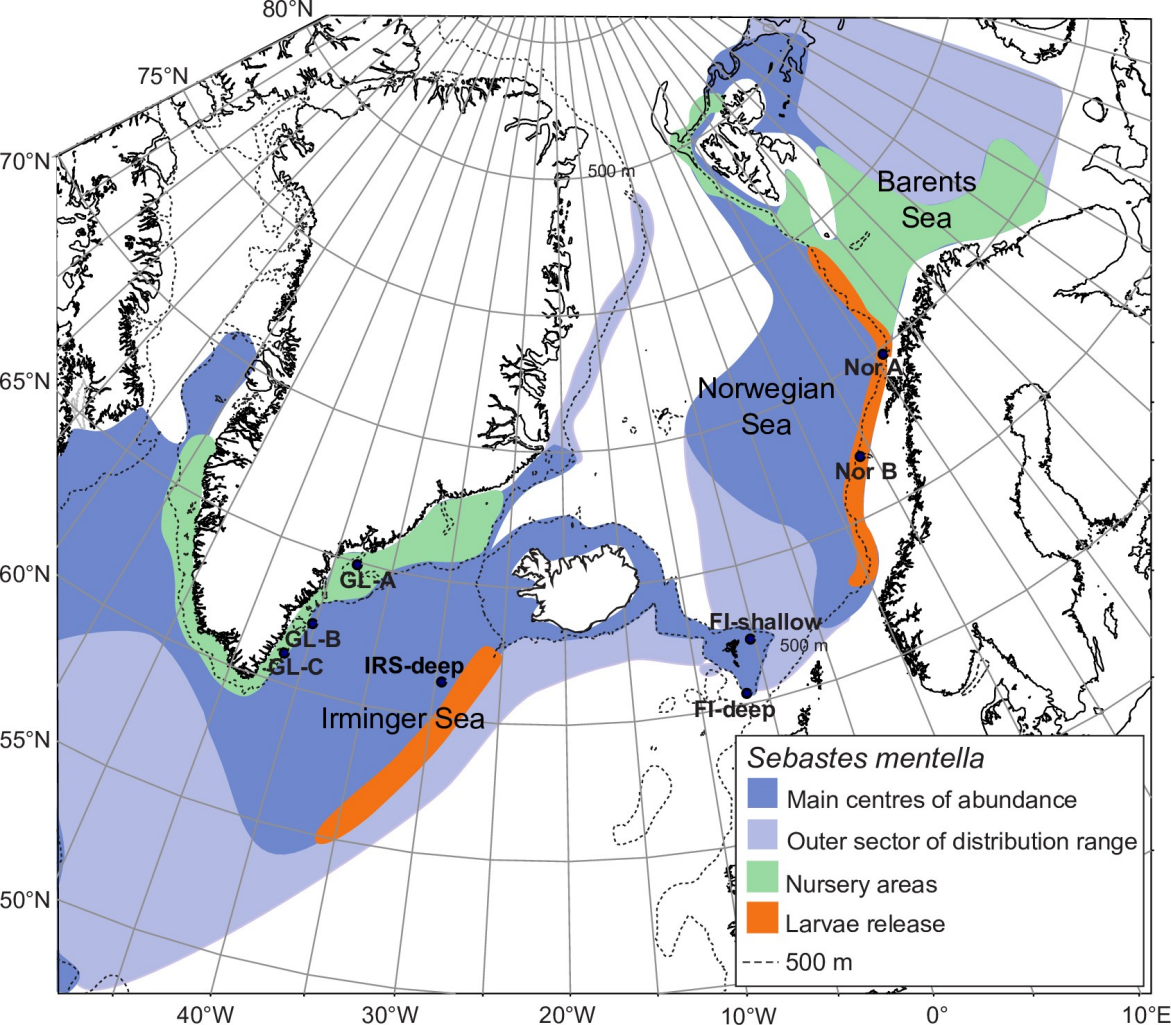
Distribution of *S*. *mentella* throughout the Northeast Atlantic (modified from Saha *et al*. [12]). Sampling locations are indicated by black circles.

One such species where cryptic intraspecific diversity has evolved to allow for colonization of multiple habitat types and thus complicating management is *Sebastes mentella* (beaked redfish), which is the most economically important species of the genus in the North Atlantic. The species has a complex life history, which has caused much debate regarding genetic population structure [6, 7]. Adult *S*. *mentella* are distributed throughout the North Atlantic and assumed to mate during winter at unknown locations. Females retain sperm for several months before the eggs are fertilized [8]. They are livebearers and release larvae in May-June in the Irminger Sea and along the Norwegian coast (Fig 1), with nursery areas off Greenland and along the Norwegian Shelf [9, 10]. Exploitation of multiple habitats by multiple populations throughout the species’ life history challenges our ability to elucidate and understand the processes involved in shaping the genetic population structure of *S*. *mentella*.

Current evidence supports three genetic groups in the Northeast Atlantic [11, 12],‘shallow (= shallow pelagic)’, ‘deep (= deep pelagic)’ and ‘slope (= demersal deep)’, which we here describe as ‘ecotypes’. The ‘shallow’ ecotype of *S*. *mentella* is the most abundant and found between 200 and 500 m water depth throughout the North Atlantic, but at 50─500 m in the Irminger Sea. The ‘deep’ ecotype inhabits waters between 550 and 800 m depth mainly in the north of the Irminger Sea. The ‘slope’ ecotype has been fished along the Icelandic shelf [11], but also found in both Greenlandic and Icelandic waters based on microsatellite DNA analysis [12]. The ‘shallow’ and ‘deep’ ecotypes differ phenotypically: the ‘deep’ ecotype has a brighter red color, stouter appearance, larger size at sexual maturity and different rates of parasite infestation [10]. The ‘slope’ fish are morphologically closer to the ‘deep’ fish [13], but they have more vertebrae and also more anal fin and pectoral fin rays compared to other *S*. *mentella* types [11, 14 for review]. Compared to the two other counterparts, the ‘slope’ ecotype seems to have much narrower distribution range. Based on microsatellite DNA and morphological data, Stefánsson et al. [15] suggested that the ‘deep’ and ‘shallow’ ecotypes represent two emerging species. DNA analyses of the mitochondrial control region, microsatellites and the gene coding for the visual-pigment rhodopsin [12, 16, 17] also indicated a deep evolutionary divergence between these two ecotypes. Divergence of *Sebastes mentella* into depth-defined habitats may have led to reproductive isolation and adaptive radiation, but this has so far not been documented using high-resolution SNP data.

Quantifying the genetic relationships among *S*. *mentella* populations is important for understanding its hierarchical genetic population structure. Allozyme studies [13, 18] indicated a closer relationship between ‘slope’ and ‘deep’ ecotypes, compared to the ‘shallow’ ecotype. Microsatellite DNA [12] suggested a closer relationship for the ‘slope-shallow’ pair compared to the ‘deep’. Furthermore, using microsatellite DNA Shum *et al*. [17] reported sub-structuring within the ‘deep’ ecotype along a longitudinal transect, but found no genetic structure within the ‘shallow’ ecotype. In contrast, Saha *et al*. [12] reported sub-structuring within both the ‘shallow’ and ‘deep’ ecotypes. Concurrent splits within the ‘shallow’ and ‘deep’ ecotypes in the Irminger Sea were suggested by Shum *et al*. [17], although no ‘slope’ ecotype was included in the study. Estimated divergence between ecotypes by microsatellites was low, thus application of high-resolution genomic markers was suggested [13] to identify neutral and adaptive genetic relationships among ecotypes.

The extent of hybridization of *S*. *mentella* is currently uncertain. Studies of allozymes and microsatellites have indicated hybridization between *S*. *mentella* and other sympatric redfish species, and between *S*. *mentella* ecotypes [19–21]. However, recent analyses using microsatellite data [13] suggested much lower hybridization between the ‘shallow’ and ‘deep’ ecotypes than the estimates by Stefansson et al [22] who also used microsatellite data. Genome wide surveys of many markers may provide more robust estimates of hybridization.

The aim of this study was to investigate genetic population structure of *S*. *mentella* by screening a large number of SNP loci by ddRAD (double digest Restriction Associated DNA). To our knowledge, this is the first SNP based investigation in *S*. *mentella*. Specifically, our objectives were: 1) Describe the genetic structure within and between the major ecotypes of *S*. *mentella*. 2) Examine across the *S*. *mentella* ecotypes for evidence of selection. 3) Estimate the extent of hybridization between the ecotypes and compare to previous microsatellite results [13]. 4) Assess the phylogenetic relationships among the *S*. *mentella* gene pools and finally 5) to develop a diagnostic SNP panel for assigning individuals to the ecotypes and populations of *S*. *mentella*.

## Materials and methods

### Sampling and DNA extraction

A total of 277 *S*. *mentella* specimens were collected by trawl from eight locations in Greenland, the Irminger Sea, the Faroe Islands and Norwegian waters during the period 1995‒2012 (Fig 1, Table 1) corresponding to eight collections. Samples of the ‘deep’ *S*. *mentella* morph were caught below 550 m depth from the Irminger Sea and Faroe Islands for the European project, REDFISH, which also provided the Faroe Islands ‘shallow’ morph specimens caught above 500 m depth. Morphological features as described by Magnusson & Magnusson [10] were included to characterize ‘shallow’ and ‘deep’ specimens. Samples from east Greenland and Norwegian (Northeast Arctic) waters were included from two different years. Total 196 of our 277 fish were the same as used in Saha et al. [12]. Adults (≥ 29 cm) and juveniles (4‒28 cm) were defined by length [23]. DNA was extracted from ethanol-preserved gill filaments using the E-Z 96 Tissue DNA Kit, following the manufacturer’s protocol (Omega Bio-Tek, Inc, Norcross, GA, USA). DNA was quantified using a broad range double-strand kit on a Qubit fluorometer (Life Technologies Corp.), and its quality was assessed by gel electrophoresis. Two collections from Norwegian waters were pooled, as Saha et al. [12] found no genetic differentiation between them.

**Table 1:** Details of the *S*. *mentella* samples analyzed.

Location	Code	Lat/Long (mean)	Time	*N*	*N*_micro_	Depth (m)	Length (cm)	Female (%)	Adult (%)	Ecotype
Northeast Arctic	Nor A*	69.38/ 15.14	Nov, 2011	20 (17)	17	575	35‒42 (40)	75	100	“shallow”
	Nor B*	66.93/ 8.17	Mar, 2009	18 (16)	16	508	33‒42 (38)	61	100	“shallow”
Faroe Islands	FI-shallow	62.78/ -6.62	Sep, 2002	38 (30)	-	415	36‒49 (44)	71	100	“shallow”
	FI-deep	60.17/ -7.84	Sep, 2002	28 (28)	-	572	43‒52 (49)	14	100	“deep”
Irminger Sea	IRS-deep	62.05/ -27.08	July, 2001	26 (14)	11	830‒850 (840)	36‒47 (42)	NA	100	“deep”
East Greenland	GL A	64.28/ -35.70	Aug, 2011	26 (26)	25	423‒430 (429)	20‒40 (32)	52	49	Mix
	GL B	61.14/ -41.46	Aug, 2011	25 (25)	26	355‒455 (425)	19‒45 (30)	46	27	Mix
	GL C	62.2/ -40.67	Aug, 2012	96 (96)	85	473‒869 (580)	28‒ 38 (34)	43	77	Mix

The sex ratio is presented as % female and life stages as % adult (rest are juveniles). Sampling depth and fish length (mean in bracket) are provided. Code = Sample code, *N* = sample size (number of fish included in the final analyses are shown in bracket), *N*_micro_ = number of fish analyzed using 13 microsatellites by Saha *et al*. [12], Ecotype = potential ecotype based on prior knowledge (mix = mixture of all three ecotypes possible) and NA = data not available (cf. Fig 1).

*The collections were pooled (renamed as ‘Nor’) for subsequent analyses, since no genetic differentiation was found by Saha *et al*. [12].

ddRAD sequencing and SNP validation

ddRAD libraries were prepared for sequencing according to the protocol by Peterson [24], using *Msp*I and *Eco*R I restriction enzymes (NEB, USA) with slight modifications including: (i) using 500 ng DNA per sample, (ii) selecting fragments from 400 to 600 bp using a Pippin-Prep 2% Agarose Gel Cassette (Sage Scientific, USA), (iii) using 12 PCR cycles and (iv) adding one clean-up cycle after the PCR step using a 1.5:1 ratio with Agencourt AMPure XP Beads (Beckman Coulter, California, USA). Libraries were validated by quantification using a Qubit® dsDNA BR Assay kit and Qubit® 2.0 Fluorometer (Invitrogen, Thermo Fisher, USA) and the size of fragments was determined using a 2100 BioAnalyzer system with DNA High Sensitivity kit (Agilent Technologies, California, USA). The mean length of fragments was 570 bp. Illumina sequencing was performed using a 500 Cycle MiSeq Reagent Kit v2 in a paired-end mode (2 x 251 bp) to produce an average of 880K (±292K) reads per individual (equivalent to 221Mb). Reads were processed to detect SNPs in STACKS software V1.18 [25]. Briefly, we used the denovo_map function with seven collections. Reads were quality checked and trimmed to a common length (240 bp) before de-multiplexing by collection. Within each collection, sequences were *de novo* clustered (allowing a maximum of 4 mismatches) to identify RAD tags. A minimum of 5 sequences were required to retain a RAD tag in an individual. We excluded loci genotyped in <80% of all fish and discarded fish genotyped at <80% of all loci. We also removed loci genotyped in <60% of fish in any collection.

Genetic diversity of the collections, the expected and observed heterozygosity (*H*_E_ and *H*_O_), minor allele frequency (MAF), inbreeding coefficient (*F*_IS_), and average number of alleles (*N*a) of the loci was estimated using the ‘*genetics*’ package in R V 3.3.1 [26]. SNPs with a MAF<0.03 over all collections were removed. *H*_E_ and *F*_IS_ of the loci were plotted against the percentage of missing data to investigate possible artifact of allele drop-out. For the final neutral and selective SNP panels, only one SNP was selected per RAD tag (described below: the one with the greatest *F*_ST_). Deviations from Hardy-Weinberg equilibrium (HWE) were tested with Fisher’s exact test using Genepop 4.2 [27] implementing the Markov Chain Monte Carlo Method (MCMC: 10,000 dememorization, 1,000 batches, and 10,000 iterations per batch). Loci deviating from HWE in all collections were removed.

### Detection of outlier SNPs

A finite-island model as implemented in BayeScan 2.1 [28] was applied for the detection of loci under selection. BayeScan attempts to identify ‘outlier’ loci by splitting *F*_ST_ into population specific components shared by all loci and locus specific components shared by all populations. We first used BayeScan to identify outliers in seven geographic collections. After the individuals were clustered into three ecotypes (but geographic collections were not pooled), we again applied BayeScan in the dataset containing 4,277 SNPs. Finally, BayeScan was used to find outlier (FDR = 0.05) in pair-wise comparisons both for the seven collections (before clustering) and three ecotypes (after clustering). For pairwise comparisons, only two collections were analyzed at a time. All sample collections were analyzed together to identify global outliers. All analyses were conducted with 20 pilot runs for 5,000 iterations, followed by 100,000 iterations with a burn-in of 50,000 steps. The default value of prior odds (10:1) was used. Loci with alpha-values significantly >0 were considered as loci under directional selection while those with alpha <0 were considered as loci under balancing selection [e.g. 29]. All other loci were considered as neutral. Convergence of BayeScan runs was assessed in R using the CODA package following Geweke’s [30] convergence diagnostics by comparing the mean of the first 10% of the MCMC chain with the mean of the last 50%. We also estimated *Gelman*.*diag* which is based on a comparison of between and within chain variances. The output of gelman.diag are the scale reduction factors for each parameter. A factor of 1 was found meaning that between- and within-chain variance are the same indicating no convergence problems.

As BayeScan is sensitive to hierarchical structure in the data, and has low power in pairwise tests, we also tested OutFLANK [31] to detect evidence of selection, although it may have very limited power in identifying spatially diversifying selection [29]. In addition, we applied Arlequin [32], which has more power but can give a higher rate of false positives [33]. The sample settings for these analyses were identical to BayeScan analyses.

To further identify regions of the genome potentially under selection, we aligned all the neutral and outlier sequences to *S*. *norvegicus* reference scaffolds [34] and to *S*. *rubrivinctus* and *S*. *nigrocinctus* scaffolds (GenBank assembly accession: GCA_000475215.1 and GCA_000475235.3, respectively) using blastn [35]. We also blasted our sequences against other eukaryote genomes in the NCBI database.

### Genetic structure

Individuals were clustered by discriminant analysis of principal components (DAPC) [36] using neutral and outlier SNPs independently in the *adegenet* R package [37]. The method uses Bayesian information criterion (BIC) to determine the optimal number of genetic clusters (K). To avoid overfitting of the discriminant functions, a limited number of PCs were used for the demonstration of the between-cluster distances in the analyses, as suggested by Jombart [37]. The function ‘*find*.*clusters*’ was applied to determine the number of clusters whereas ‘*optima*.*a*.*score*’ was used to estimate the optimal number of PCs to retain. Finally, the identified clusters were named as the ‘shallow’, ‘deep’ and ‘slope’ ecotypes depending on the occurrence of reference fish, and were compared to the clusters identified by Saha et al. [12].

In an effort to identify possible hybrids between the ecotypes, a Bayesian clustering approach, as implemented in STRUCTURE 2.3.4 [38], was used for clustering of genotypes and estimation of individual admixture proportion. STRUCTURE was run for a K of one to five, both for neutral and outlier SNP panels, with five replications for each K, using the model for admixture ancestry and correlated allele frequencies. We used 200,000 burn-in iterations and 200,000 MCMC steps for the analyses. Finally, the delta K Evanno criterion [39] was plotted to detect the optimal number of genetic clusters (K) best describing the datasets. We also used mean LnP(K) values from STRUCTURE to determine the number of genetic clusters. Hybrids were identified as individuals with *q* values between 0.1 and 0.9 [40, 41]. We also compared the observed *q* values based on microsatellite and SNP data for the 96 ‘shallow’ and ‘deep’ fish from Greenland waters.

Population divergences, before and after clustering (see Results), were measured by pairwise *F*_ST_ [42] and tested with 10,000 permutations in Arlequin 3.5.1.3 [32]. Arlequin was also used to perform a hierarchical AMOVA (analysis of molecular variance) using both neutral and outlier SNP panels. A three-ecotype configuration based on clustering outputs (without pooling the geographical collections) was used for the AMOVA. We applied a false discovery rate control [FDR, 43] to minimize type I error associated with multiple pairwise comparisons.

### Inference of evolutionary scenarios of *S*. *mentella* ecotypes

The population history of the ecotypes were inferred by Approximate Bayesian Computation (ABC) in DIYABC V2.1.0 [44]. The ABC method infers the posterior distribution of model parameters (here, population size parameters, N1 to N5, and time since divergence, t1 to t3) using summary statistics from simulations [44]. Since no differentiation between ‘shallow’ fish from the Faroe Islands and Norwegian waters was found (see results), the ‘shallow’ collection from the Faroe Islands water was excluded in this analysis. The DAPC suggested that ‘deep’ fish from Greenland waters (GL-deep) were mixture of ‘deep’ fish from the Faroe Islands and Irminger Sea waters (see results) and therefore the ‘GL-deep’ collection was also excluded from the analysis.

To reduce the number and complexity of possible scenarios, we ran a two-stage DIYABC analysis. In stage-1, our objective was to assess the genetic relationship of the ‘shallow’ and ‘deep’ *S*. *mentella* gene pools and to examine if a scenario similar to that of Shum *et al*. [17, S1 File] was supported by SNP data. Here, we compared six alternative evolutionary scenarios for the four gene pools similar to those of Shum *et al*. [17] (S1 File). Scenario-1 proposes the original split between the ‘shallow’ and ‘deep’ groups, followed by split between the Irminger and Faroe seas within the ‘deep’ group and between Greenlandic and Norwegian waters within the ‘shallow’ group. Scenario-2 suggests independent origin of the ‘deep’ groups from their ‘shallow’ ancestors in the Norwegian and Greenlandic waters. Unlike scenario-2, scenario-3 has an independent origin of the ‘shallow’ groups from their ‘deep’ group ancestors. In scenarios 4, 5 and 6, a step-wise divergence was proposed at t1, t2 and t3. Once the genetic relationship of the ‘shallow’ and ‘deep’ *S*. *mentella* gene pools was determined, we added the ‘slope’ ecotype in the analysis and proceeded with stage-2 to determine the complete scenario (S1 File). In stage-2, scenario-1 proposes first split between the ‘deep’ and ‘shallow’ groups followed by the second split between the ‘shallow’ and ‘slope’ groups. In scenario-2, the second split occurs between the ‘deep’ and ‘slope’ groups. Scenario-3 suggests a concurrent split among the ‘shallow’, ‘deep’ and ‘slope’ groups.

Of the total 1,943 neutral SNPs identified by the BayeScan analysis (see result), only those with an overall MAF≥0.05 (632 SNPs) were used. Each scenario was given uniform prior probability (10–10,000) and 32 summary statistics for genic diversities, *F*_ST_ and Nei’s distances (S1 File, also see for further details of the analysis) were selected to generate reference tables containing 10^6^ simulated datasets per scenario. We then estimated the relative posterior probability for each scenario using the *direct* and *logistic regression* methods on 0.1 and 1% of the simulations closest to the observed data set, respectively.

### Identification of discriminatory SNPs by random forest analyses

We used Random Forest [45] to identify an informative SNP panel with reduced number of loci to discriminate among the ‘shallow’, ‘deep’, and ‘slope’ ecotypes and their populations of *S*. *mentella*. As suggested by Anderson et al [46], we used a training dataset containing 50% of the total fish to identify discriminatory loci, and the remaining 50% fish as a test dataset to estimate assignment accuracy. Random forest is a machine learning algorithm that identifies loci that best differentiate ecotypes using a “forest” of decision trees [45]. Here, we used the *randomForest* package in R [47] and a two-step process to identify the marker panel. First, an estimate of the importance of each locus for ecotype differentiation was estimated by using three random forest analyses. Of the five genetic populations supported by the DAPC method (‘Nor-shallow’, ‘GL-shallow’, ‘FI-deep’, ‘IRS-deep’ and ‘GL-slope’; see result), we only excluded the ‘shallow’ population of Greenland from this analysis because of its small sample size (n = 17). For each analysis, 31, 41, 21 and 21 specimens from the ‘Nor-shallow’, ‘FI-deep’, ‘IRS-deep’ and ‘GL-slope’ populations (50% of the available individuals = training dataset) were used to create 10,000 trees using all (n = 2,040) predictor loci. Markers were ordered by their average importance score and the set of loci with the lowest out-of-bag error rate were retained for further analysis. Second, the subset of the most important loci were further reduced using a backward purging approach [48]. In this step, the least important loci were sequentially removed from the analysis and a new out-of-bag error rate was estimated until only two loci remained in the data set. Three random forest analyses were performed for each set of loci, and the group of loci with the lowest out-of-bag error rate was retained for the population identification. The efficacy of the identified marker panel was evaluated using the *predict* function with the random forest model and 31, 41, 21 and 20 additional specimens from the ‘Nor-shallow’, ‘FI-deep’, ‘IRS-deep’ and ‘GL-slope’ populations, i.e. remaining 50% of the available individuals from each population.

## Results

### ddRAD sequencing and SNP validation

Analysis of sequencing data generated 68,491 RAD tags, of which 19,005 contained one or more SNPs. Of the total 42,288 SNPs, 27,876 (66%) were excluded due to excessive missing data (>20%), retaining 14,412 SNPs. We removed 25 fish with incomplete genotypes in >20% of loci, retaining 252 fish (Table 1). A total of 3,740 SNPs were discarded because of missing data in >60% fish in any collection. After removing SNPs with MAF<0.03 in all collections, 4,378 SNPs remained (S2 File). Of the total 30,646 tests for HWE, 1,321 tests (4.31%) showed deviation (P <0.05). Only 155 tests remained significant after FDR control, 119 of which showed deviation in a collection from Greenland waters (GL C). Five SNPs were removed since they deviated from HWE in all collections (S2 File).

Neither individual multilocus heterozygosity nor locus-specific *F*_IS_ (S3 File) was correlated with the percentage of missing data (R^2^ = 0.01 and 0.07, respectively). However, 139 SNPs had *F*_IS_>0.60 or *F*_IS_<-0.60. Ninety-six of those loci were homozygous in three to six of the seven collections and had MAF<0.05 in one to four other collections, and so were excluded from the dataset. At that stage, we retained 4,277 SNPs in 2,040 RAD tags (genome scan methods for finding outliers were applied in this dataset, see below). The mean sequencing depth for the 2,040 RAD tags was 21 (S3 File). The mean *H*_E_ and *F*_IS_ of the loci over collections ranged from 0.143 to 0.203, and 0.046 to 0.096, respectively (S3 File). There was no correlation between locus specific *F*_IS_ and *H*_E_ (S3 File). After the identification of 97 outlier loci (described below), we selected one SNP from each of the 1,943 RAD tags (the one with the greatest *F*_ST_) as a final dataset. Descriptive statistics for each selected locus are available in S3 File.

### Outlier SNPs

**Fig 2:**
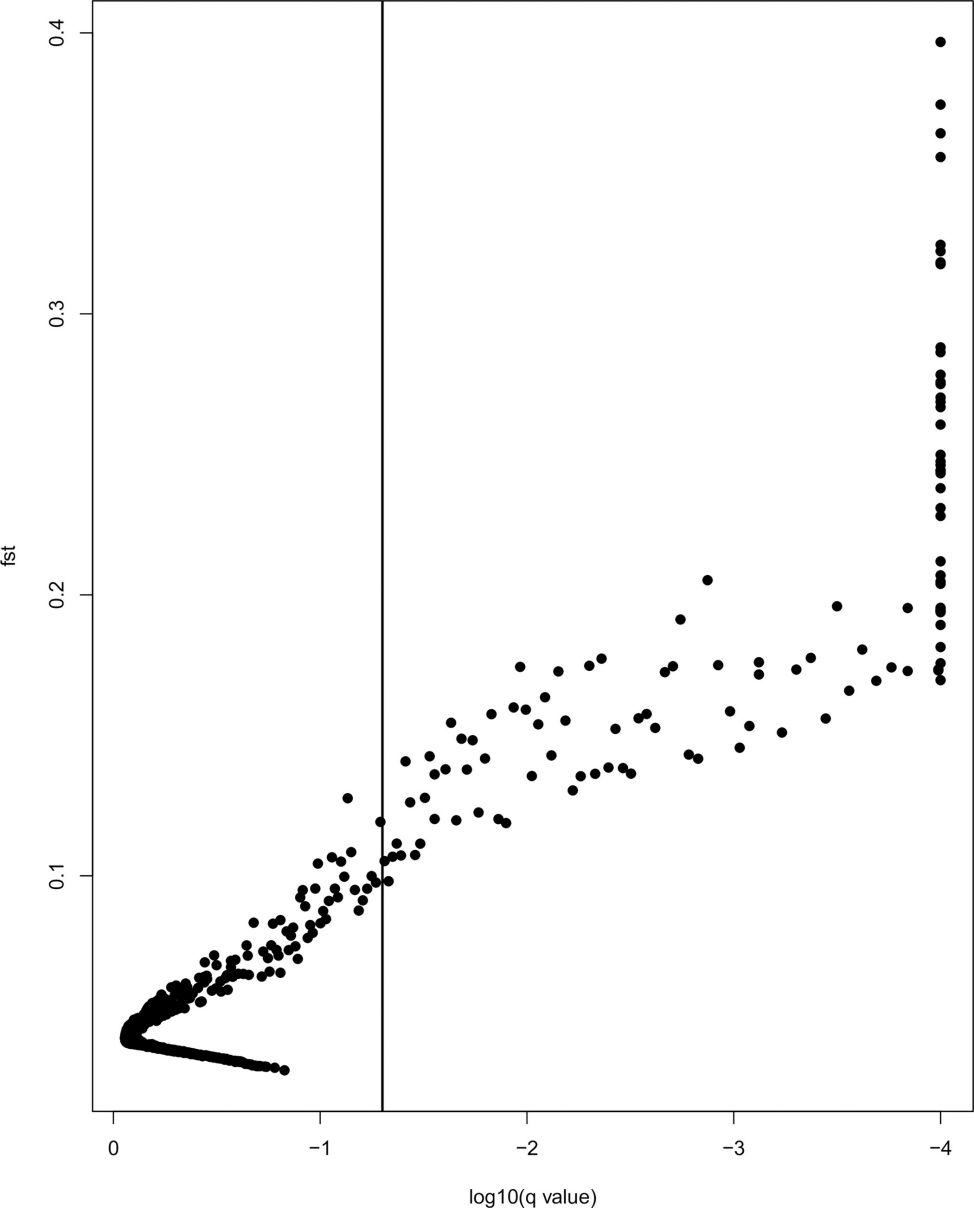
Results from the BayeScan analysis illustrating neutral and selective SNPs. The vertical line represents the log_10_ (q-value) corresponding to an FDR = 0.05, to the right of which 109 potentially loci under selection are shown.

Among the seven collections (before clustering), BayeScan detected 90 SNPs under directional selection in the dataset containing 4,277 SNPs (FDR = 0.05). After clustering of individuals into three ecotypes (“shallow”, “deep”, and “slope”), BayeScan identified in total 109 outliers from 4,277 SNPs (Fig 2). No outliers were identified in any pairwise comparisons. OutFLANK found no outliers (not shown), while Arlequin identified 407 outliers (72 overlapped with BayeScan output, results not shown). The 109 outliers from BayeScan came from 97 RAD tags (hereafter we only present outliers from BayeScan), of which 60 RAD tags had both neutral (none of which were included in the neutral panel) and outlier SNPs. Thirty-seven outlier RAD tags had only one SNP each, while seven RAD tags had 2─4 outlier SNPs each. We selected one outlier per RAD tag, resulting in 97 outliers. Sixty loci appeared in both outlier SNP panels (based on data before and after clustering). For the subsequent analyses, we included 97 outlier SNPs coming from 97 RAD tags in the outlier panel.

In total, 82 RAD tags (containing one outlier each) aligned to Atlantic *Sebastes* scaffolds (*S*. *norvegicus*: 75,627 scaffolds*)* while 92 and 89 RAD tags aligned to Pacific (*S*. *rubrivinctus*: 68,206 scaffolds and *S*. *nigrocinctus*: 15,872 scaffolds, respectively) *Sebastes* scaffolds (E-score = 7.00X10^-40^, S4 File). Two RAD tags aligned to the same scaffold in *S*. *nigrocinctus* genome and also in *S*. *norvegicus* genome, four other RAD tags aligned to two scaffolds in *S*. *norvegicus* genome (two in each) and two RAD tags aligned to the same scaffold in *S*. *rubrivinctus* genome (meaning that there were total eight RAD tags sharing scaffolds with one or more outliers).

The findings that the neutral and selective SNPs are located further apart in the genome, and that some selective SNPs are close to one another may imply selective sweep and/or genetic hitchhiking in the genome, so we investigated proximity of neutral vs selective SNPs. Total 1,792, 1,912 and 1,714 RAD tags containing neutral SNPs aligned to *S*. *norvegicus*, *S*. *rubrivinctus* and *S*. *nigrocinctus* scaffolds, respectively (E-score = 5.00X^-21^, S4 File). Four RAD tags containing neutral SNPs aligned to the same scaffold as outliers in *S*. *norvegicus* genome. There were thirteen and three such RAD tags that aligned to the same scaffold as outliers in *S*. *rubrivinctus* and *S*. *nigrocinctus* genomes, respectively. Interestingly, no neutral loci were identified in the same scaffold as any of the eight mentioned outliers (sharing scaffolds with one or more other outliers). Of the 97 outliers, only 15 aligned to the same scaffold as neutral SNPs (S4 File), while total 233 scaffolds had two or more neutral SNPs.

Alignment of the 97 outlier sequences to other genomes in the NCBI database provided significant hits for 43 SNPs (S5 File). Nine hits were associated with genes involved in growth and/ or sexual developmental functions in fish, e.g. *Sebastes schlegelii*, *Dicentrarchus labrax*, *Salmo salar*. Eleven hits were associated with genes providing immunological responses in fish, e.g. *Oplegnathus fasciatus*, *Maylandia zebra*, *Lates calcarifer* and *Dicentrarchus labrax*. Interestingly, some of these outliers aligned to the same scaffold (S5 File), such as six RAD tags aligned to two scaffolds of *Dicentrarchus labrax*. Furthermore, most of these loci were identified on the same end of the chromosomes. Other sequences containing outlier SNPs were predicted as linked with complex cellular and molecular functions.

### Pattern of genetic structure and connectivity

**Fig 3:**
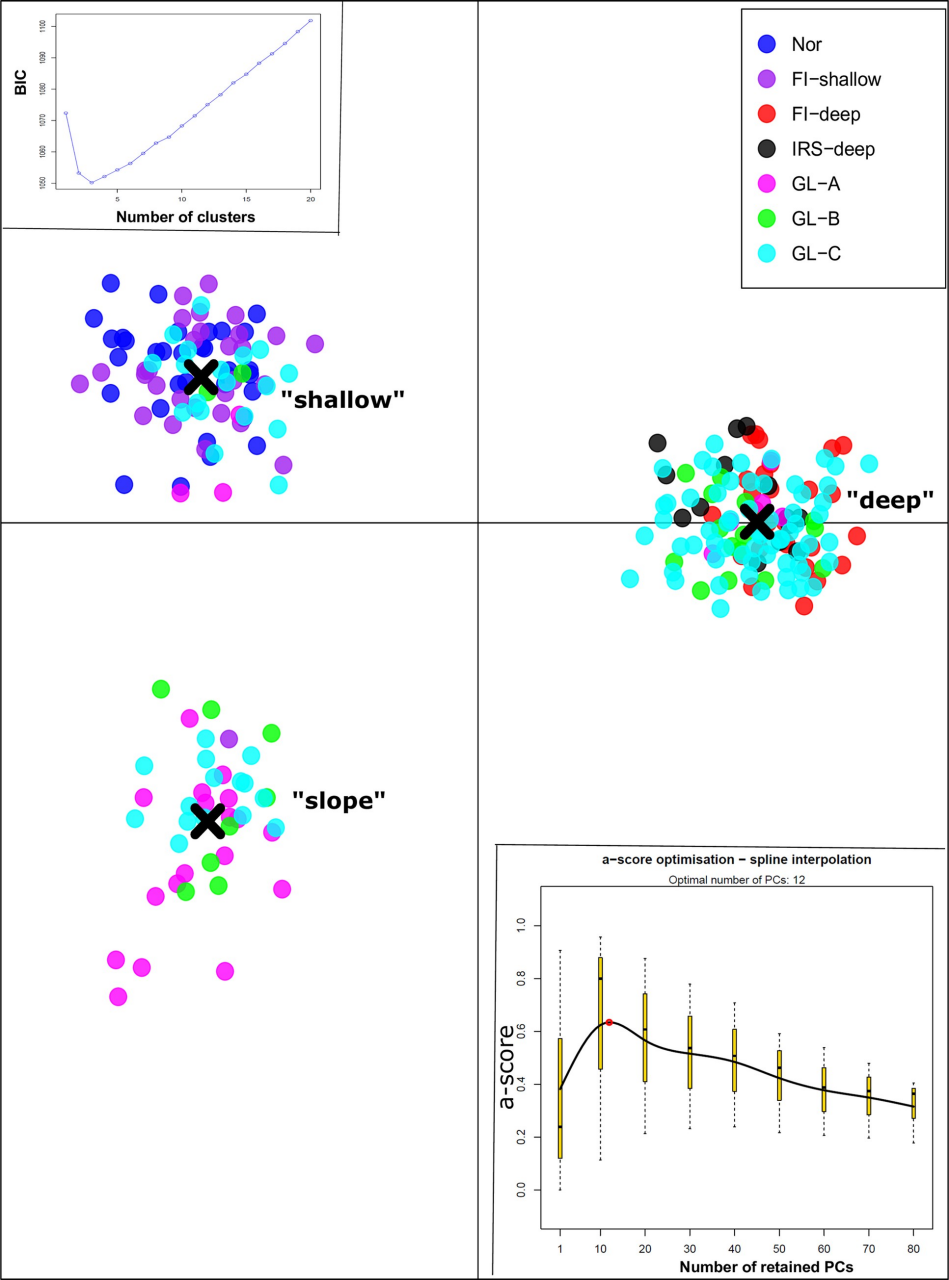
Discriminant analysis of principal component (DAPC) analysis of *S*. *mentella* samples using 1,943 neutral SNPs. BIC value supported three clusters for the given dataset. Twelve PCs and two discriminant functions were retained. Clustering of individuals from different collections are presented.

Using data from 1,943 neutral SNPs, the DAPC suggested three clusters of *S*. *mentella* (Fig 3). We retained 12 PCs and two discriminant functions to represent the between-cluster structures. The ‘shallow’ and ‘deep’ samples from the Faroe Islands and Irminger Sea waters appeared non-mixed, except for one individual from the Faroe Islands which was sampled as the ‘shallow’ ecotype but clustered with the ‘slope’ ecotype (described below). All the fish from Norwegian waters clustered with the ‘shallow’ ecotype. In contrast, the collections from Greenland appeared to be highly mixed, as was expected from the results of HWE tests and the high *F*_IS_ values (Table 2) indicating a Wahlund effect. These samples were represented in all three clusters: Twenty-three fish (15 adults and 8 juveniles) clustered with the ‘shallow’ ecotype, while 83 (71 adults and 12 juveniles) with the ‘deep’ ecotype. The third cluster mainly consisted of fish from Greenland waters (35 adult and 6 juvenile fish) but also of one fish from the Faroe Islands and total 34 of these fish were previously identified as the ‘slope’ ecotype fish by Saha et al. [12]. Using neutral SNPs, DAPC did not identify further structure within ‘deep’ or ‘shallow’ collections when analysed separately.

**Table 2:** Descriptive statistics for *S*. *mentella* collections based on the selected 1,943 neutral and 97 outlier SNPs.

	1943 neutral SNPs			97 outlier SNPs		
Sample	*H*_O_	*H*_E_	*F*_IS_ (95% CI)	*H*_O_	*H*_E_	*F*_IS_ (95% CI)
Nor	0.185	0.193	0.041 (0.039–0.044)	0.243	0.261	0.069 (0.067–0.071)
FI-shallow	0.189	0.203	0.070 (0.067–0.071)	0.243	0.278	0.126 (0.123–0.129)
FI-deep	0.185	0.195	0.051 (0.049–0.054)	0.305	0.341	0.106 (0.103–0.108)
IRS-deep	0.191	0.201	0.050 (0.046–0.053)	0.344	0.380	0.095 (0.087–0.103)
GL A	0.190	0.204	0.069 (0.066–0.071)	0.235	0.282	0.167 (0.163–0.170)
GL B	0.170	0.188	0.096 (0.093–0.098)	0.277	0.332	0.166 (0.162–0.170)
GL C	0.130	0.142	0.085 (0.084–0.085)	0.249	0.321	0.224 (0.222–0.227)

*H*_O_ = Observed heterozygosity, *H*_E_ = Expected heterozygosity, and *F*_IS_ = Inbreeding coefficient (values within 95% CI are presented).

**Fig 4:**
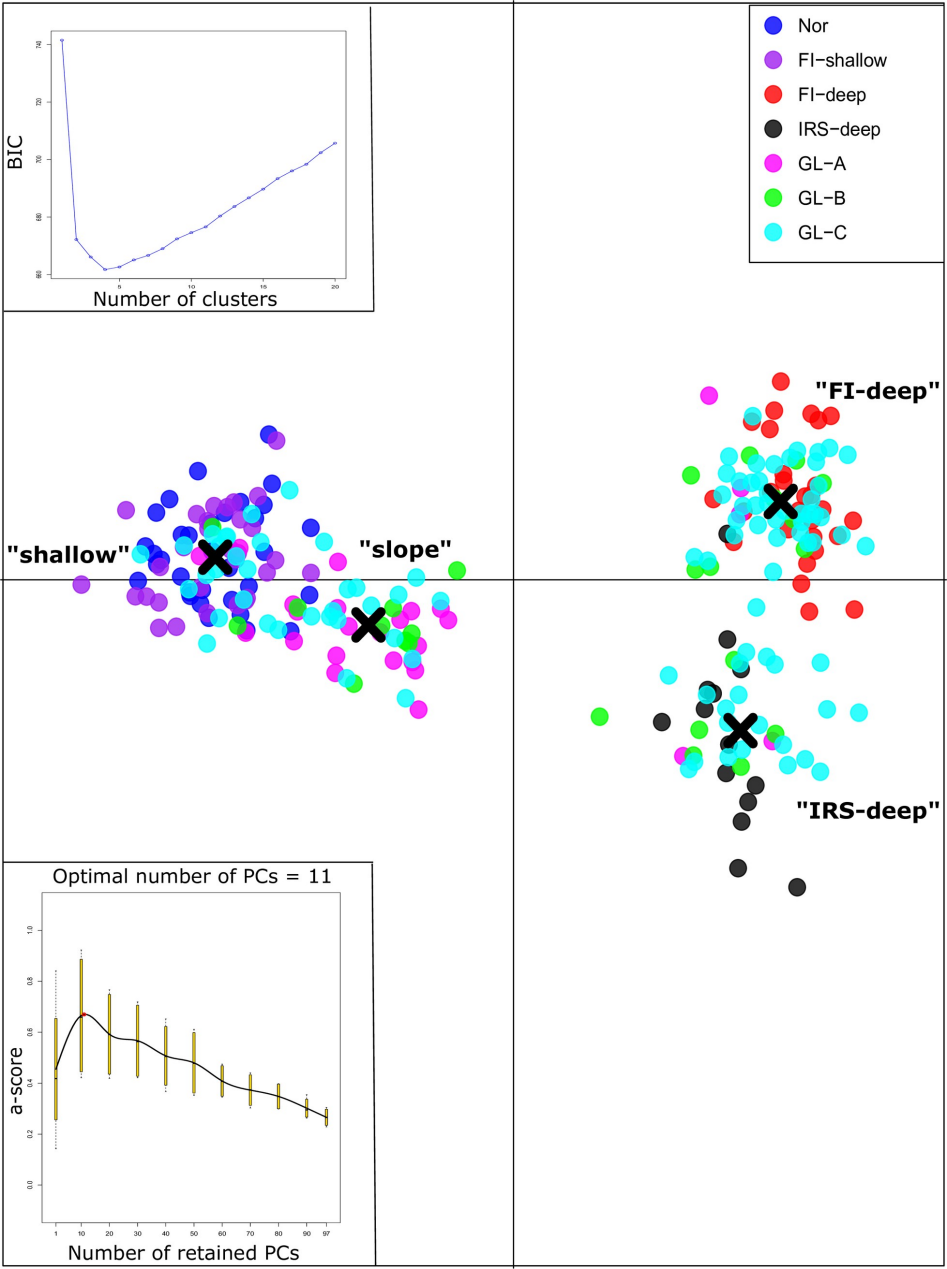
DAPC analysis of *S*. *mentella* samples using 97 outlier SNPs. BIC value supported four clusters for the given dataset. Eleven PCs and three discriminant functions were retained. Clustering of individuals from different collections are presented.

Using data from 97 putatively adaptive SNPs, the DAPC supported four genetic clusters of *S*. *mentella* (Fig 4). We retained eleven PCs and three discriminant functions to represent the between-cluster structures. Compared to the results from analysis of neutral SNPs, there was no substantial change in composition of the ‘shallow’ and ‘slope’ ecotypes in the results from analysis of outlier SNPs. Only five fish, from Greenlandic waters, clustered differently between the ‘shallow’ and ‘slope’ ecotypes. Data from outlier SNPs supported two clusters within the ‘deep’ collections, both when all samples and only ‘deep’ samples were analyzed. One of the ‘deep’ clusters included all the reference ‘deep’ ecotype fish from the Faroe Islands waters and was called ‘FI-deep’. The ‘FI-deep’ cluster also included one fish from the Irminger Sea and 56 fish from Greenland waters. Another ‘deep’ cluster included all but one ‘deep’ ecotype fish from the Irminger Sea and was called ‘IRS-deep’. The ‘IRS-deep’ cluster included 26 fish from Greenlandic waters. When only the ‘shallow’ samples were analysed by DAPC using 97 outliers, two populations were supported (not shown). One population including fish from the Faroe Islands (east) and Norwegian waters plus six fish from Greenland, another population included only fish from Greenland (n = 17).

STRUCTURE suggested only two genetic clusters for our samples (using both Evanno and mean LnP(K) methods), using data from both the neutral and outlier SNP panels (S6 File). The main difference between the clustering outputs from the DAPC and STRUCTURE was that the DAPC identified a distinctive ‘slope’ cluster, whereas STRUCTURE placed all ‘slope’ fish within the ‘shallow’ ecotype.

The AMOVA supported the three-ecotype configuration (“shallow-deep-slope”) of the *S*. *mentella* samples using data from both neutral (*F*_CT_ = 0.05, *P* = 0.014) and outlier (*F*_CT_ = 0.31, *P* < 0.001) SNPs. Likewise, pairwise *F*_ST_ estimates were highly significant among the three ecotypes (Table 3). Differentiation was the largest between ‘deep’ and ‘slope’ for neutral markers, but between ‘deep’ and ‘shallow’ for outlier SNPs.

**Table 3:** Pair-wise *F*_ST_ values between the “shallow”, “deep” and “slope” ecotypes of *S*. *mentella* identified by DAPC analyses.

	shallow	deep	slope
shallow	**85**	0.327 (97)	0.119 (43)
deep	0.058 (776)	**125**	0.238 (79)
slope	0.037 (340)	0.063 (557)	**42**

*P* < 0.00001 for all comparisons. *F*_ST_ estimates using 1,943 neutral (below diagonal) and 97 outlier (above diagonal) SNPs are shown.

Collection sizes are presented along the diagonal.

Number of SNPs estimating significant *F*_ST_ (*P* < 0.05) values between the ecotypes are shown in brackets.

A lower, but significant proportion of the genetic variance could be ascribed to divergence among populations within ecotypes using both neutral (*F*_SC_ = 0.008, *P* < 0.00001) and outlier (*F*_SC_ = 0.039, *P* < 0.00001) SNPs. Using neutral SNPs, no differentiation between the Faroe Islands (FI-shallow) and Norwegian (Nor-shallow) or Greenland ‘shallow’ was apparent (Table 4). Nevertheless, ‘shallow’ fish from Norwegian waters differentiated from Greenlandic fish. Collections of the ‘deep’ ecotype from the Faroe Islands (FI-deep), Irminger Sea (IRS-deep), and Greenland (GL-deep) waters were significantly differentiated. When *F*_ST_ was estimated using the outliers, all collections were differentiated from one another, except the ‘shallow’ fish from the Faroe Islands and Norway.

**Table 4:** Pair-wise *F*_ST_ values between different collection pairs of *S*. *mentella* after clustering by DAPC analyses.

	*N*	Nor-shallow	FI-shallow	GL-shallow	FI-deep	IRS-deep	GL-deep	GL-slope
Nor-shallow	33	-	**-0.011**	0.048	0.383	0.387	0.319	0.139
FI-shallow	29	**-0.008**	-	0.038	0.384	0.388	0.324	0.127
GL-shallow	23	0.002	**0.002**	-	0.351	0.341	0.300	0.109
FI-deep	28	0.069	0.063	0.066	-	0.102	0.016	0.292
IRS-deep	14	0.070	0.055	0.059	0.018	-	0.053	0.313
GL-deep	83	0.060	0.054	0.054	0.005	0.010	-	0.237
GL-slope	42	0.037	0.035	0.036	0.072	0.071	0.062	-

The ‘shallow’, ‘deep’ and ‘slope’ ecotypes identified from different sampling locations (cf. Table 1) are compared. *F*_ST_ estimates using 1,943 neutral (below diagonal) and 97 outlier (above diagonal) SNPs are presented. Values except those in bold are statistically significant (*P* ≤ 0.05) even after FDR (= 0.05) control. *N* = collection sizes.

**Fig 5:**
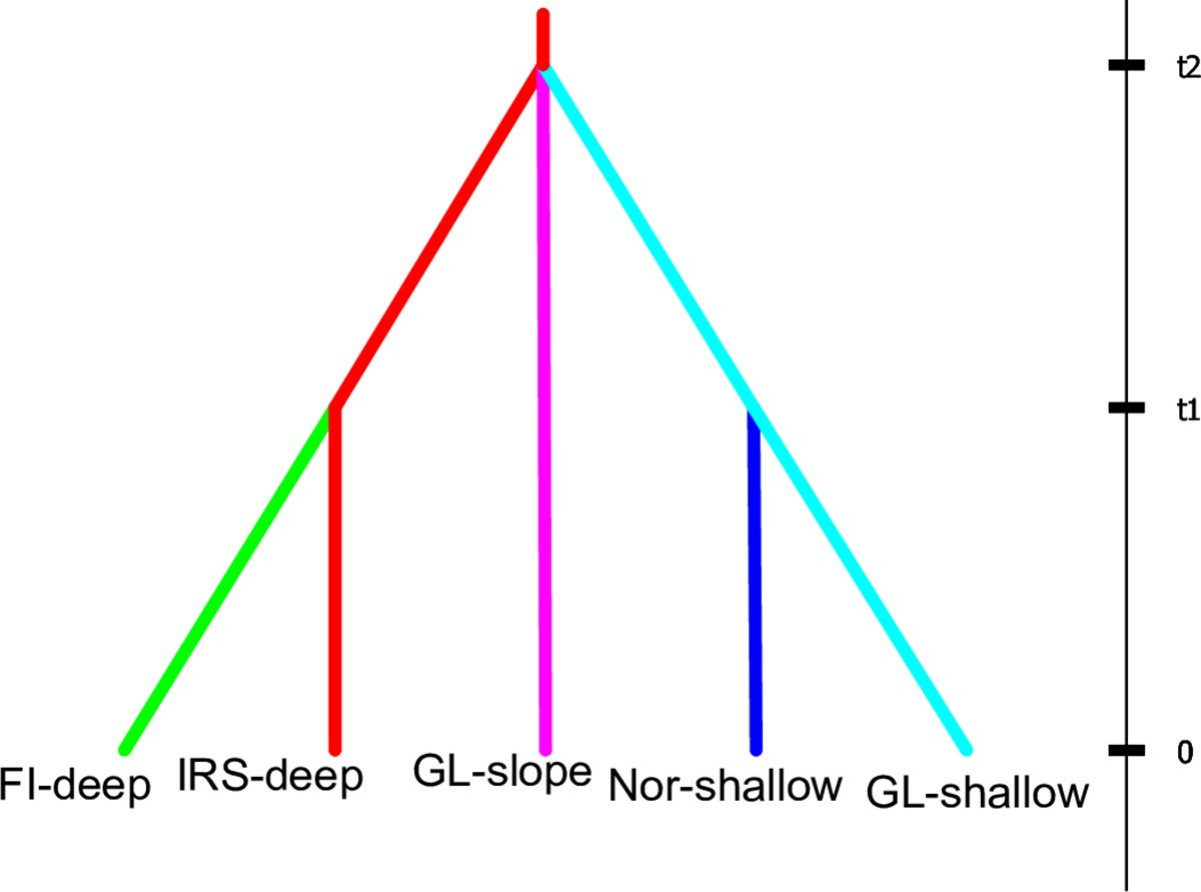
Results from the DIYABC analysis supporting a concurrent split among the ‘shallow’, ‘deep’ and ‘slope’ ecotypes. Subsequent splits were estimated within the ‘shallow’ and ‘deep’ ecotypes (for details see S1 File).

DIYABC, using both *direct* and *logistic regression* methods in stage-1 of analysis, supported an original split between the ‘shallow’ and ‘deep’ ecotypes, followed by split between the Irminger and Faroe seas within the ‘deep’ ecotype and between Greenlandic and Norwegian waters within the ‘shallow’ ecotype (Fig 5, S1 File). In stage-2, a concurrent split was supported among the ‘shallow’, ‘deep’ and ‘slope’ ecotypes 602 generations ago (Fig 5, S1 File; 95% CI = 288─932). Subsequent splits within both the ‘shallow’ and ‘deep’ ecotypes were estimated 150 generations ago (95% CI = 51.3─302). The ‘shallow’ ecotype in Norwegian waters was supported to have emerged from the ‘shallow’ ecotype in Greenland waters.

### Hybridization based on genome-wide SNPs vs. microsatellites

A total of 180 fish were analyzed both in the present investigation and the microsatellite study by Saha et al. [12]. For a threshold *q* value between 0.1 and 0.9, Saha et al. [12] identified 28 of these fish as ‘hybrids’ (S7 File). STRUCTURE based on SNP data identified eleven fish as hybrids for *q* between 0.1 and 0.9, only three of which were previously detected with microsatellite data, whereas eight hybrids were supported as ‘purebreds’ (*q*>0.97) by microsatellites. When the observed *q* values based on microsatellites and SNP data for the ‘shallow’ and ‘deep’ ecotypes from Greenland waters were compared, microsatellite data provided more ‘intermediate’ *q* values (S7 File).

### Selection of a highly discriminatory SNP panel

Random Forest analyses suggested a SNP panel with only 21 loci discriminating the ecotypes and their populations, which was retained for the genetic population identification. The *predict* function of the model estimated that using 21 discriminatory SNPs 94%, 95%, 95% and 91% of the test individuals from the ‘Nor-shallow’, ‘FI-deep’, ‘IRS-deep’ and ‘GL-slope’ populations, respectively, assigned to their true ecotypes and populations (S8 File).

## Discussion

We applied ddRAD sequencing in *S*. *mentella* to understand effects of the complex biology and ecology on the population genetic structure of the species identifying over 2,000 SNPs including neutral and outlier loci following stringent SNP filtering. Neutral loci clearly distinguished among the ‘shallow’, ‘deep’ and ‘slope’ ecotypes. The outliers suggested evidence for local adaptation and identified genetic structure at a finer spatial scale. Genome-wide SNP markers provided inferences of the population genetic structure of *S*. *mentella* and a robust description of its complex genetic structure. We reported a discriminatory SNP panel of only 21 SNPs that can assign fish back to the *S*. *mentella* ecotypes and their populations with a high precision. A cost-effective set of SNP markers is vital for monitoring the population in fisheries management [49, 50]. Our SNP panel is expected to complement morphological data, as visual discrimination among the *S*. *mentella* ecotypes is difficult.

### Population genetic structure

Divergence among the three ecotypes clearly supports existence of the ‘shallow’ ecotype throughout the Northeast Atlantic, the ‘deep’ ecotype in the Faroe Islands, Irminger Sea and Greenland waters, and the ‘slope’ ecotype in Greenland waters. The occurrence of the ‘shallow’ and ‘deep’ ecotypes are in agreement with the finding using hemoglobin and allozyme marker systems [51], microsatellites [12, 16, 22], mitochondrial DNA [16, 17] and morphological data [10]. The identification of the ‘slope’ ecotype is consistent with results based on microsatellite [12, 22] although Stefansson et al [22] only studied this ecotype at the Iceland shelf.

Genome-wide SNPs supported fine scale sub-structuring within the ‘shallow’ and ‘deep’ ecotypes (Table 4, Fig 4). The sub-structuring was highly pronounced within the ‘deep’ ecotype, with collections from the Faroe Islands, Irminger Sea, and Greenland waters significantly differentiated from one another. The clustering based on outlier SNPs supported only populations at the Faroe Islands and Irminger Sea, while the ‘deep’ ecotype from Greenland waters assigned to both populations (Fig 4). This mixture in Greenlandic waters strongly supports a nursing area for both the ‘deep’ ecotype populations in Greenland [8, 13]. Differentiation between the Faroe Islands and Irminger Sea ‘deep’ collections supports the notion by Shum *et al*. [17] that fish from the ‘deep’ ecotype may be less migratory than fish from the ‘shallow’ ecotype, and /or lower divergence within the ‘shallow’ ecotype is linked with their more recent evolutionary background as reflected by our DIYABC estimates. The ‘deep’ ecotype showed divergence between close neighboring ridges, i.e. the Reykjanes and Faroe Island ridges, isolated by deeper ocean and possibly associated with complex oceanic currents at depth [17]. Similar observation was reported for other species from the area such as saithe [52].

Our data clearly supported an isolated genetic population within the ‘shallow’ *S*. *mentella* in the Northeast Arctic (Norwegian) waters, which also includes ‘shallow’ fish north of the Faroe Islands. Roques *et al*. [19] found one Norwegian collection differentiated from the pan-oceanic ecotype, whereas other studies described the ‘shallow’ ecotype as homogenous across the North Atlantic [17, 22]. Saha et al. [12] suggested a separate Northeast Arctic population within the ‘shallow’ *S*. *mentella*. A distinctive Northeast Arctic population within the ‘shallow’ ecotype is supported by the existence of independent larval extrusion and nursery grounds along the Norwegian shelf [8, 9, 13]. In line with our observation, Chelak *et al*. [53] found no differentiation between ‘shallow’ fish from the Faroe Islands (north) and those from Norwegian waters.

Our SNP data strongly supported the presence of ‘slope’ fish in the continental slope off east Greenland. The ‘slope’ ecotype identified by Stefansson *et al*. [22] in Icelandic waters is likely similar to the ‘slope’ ecotype reported by Saha et al. [12] as they both used Icelandic shelf slope fish as reference from the same project REDFISH [13, 18].

One ‘shallow’ fish from the Faroe Islands water clustered with the ‘slope’ ecotype in the present study (Figs 3 and 4). In the investigation by Saha et al. [12], few fish from Norwegian waters clustered with the ‘slope’ ecotype, and one was identified as a ‘shallow’ fish in this study. Although the ‘slope’ fish have been suspected to occur in waters around the Faroe Islands, no such indications exist for Norwegian waters [11]. It is therefore possible that STRUCTURE [38] misidentified a few fish from Norwegian waters as ‘slope’ fish for the microsatellites [13]. The program has been found to miss differences among low *F*_ST_ marine fish populations [54] and misclassify individuals when genetic differentiation among populations is low [55].

### Adaptive divergence?

Results from the analyses of genome-wide SNPs suggested adaptive divergence in the *S*. *mentella* complex. We identified in total 109 outliers in our dataset of seven geographical collections belonging to the three ecotypes of *S*. *mentella*. In line with the results based on neutral SNPs, the outliers precisely differentiated the ‘shallow’, ‘deep’ and ‘slope’ ecotypes of the species. It is therefore possible that along with genetic drift, adaptive processes are at play within the species complex, also suspected by Cadrin *et al*. [11], Shum *et al*. [17] and Shum *et al*. [16]. The reason for the lack of outliers in pair-wise comparisons of samples may be the lower power of BayeScan for comparison with few population [33].

Alignment analyses of the tentative adaptive sequences revealed that many outliers are linked with functional parts of the genome serving immunology, growth and/or sexual development, as well as complex cellular and molecular functions. Fish from the ‘deep’ ecotype are known to be larger in size at sexual maturity and have less parasitic infestation compared to their ‘shallow’ counterparts [see 11], and outliers may therefore represent *S*. *mentella* genomic region under selection. Similar divergence in immune related genes has been revealed in Atlantic salmon [56] and stickleback [57]. More specifically, signatures of parasite-driven selection have been reported in Atlantic salmon [58].

A selective sweep in *S*. *mentella* genome was indicated, as some of our outliers were on same scaffolds of the *Sebastes* genomes and multiple outliers aligned to the same locations of the *Dicentrarchus labrax* genome. Furthermore, these scaffolds contained no neutral loci and only a few outliers aligned to the same scaffold as neutral SNPs (S4 File). These patterns indicate hitchhiking selection [59, 60] and support the notion of selective divergence. In Atlantic salmon, multiple selective sweeps linked with immunological functions have been identified on different chromosomes [56]; however, more genomic information is required to support this hypothesis in *S*. *mentella*. Alternatively, it is possible that the observed genomic divergence is linked with chromosomal inversions as suggested for Atlantic cod [61].

There are many caveats in inferring selection from outlier analyses. Methods implemented in Arlequin or OutFlank have been suggested to perform better in hierarchical data like ours [31, 62]. However, a simulation study found high false positive rates for Arlequin [63], while OutFlank was shown to have low power for identifying spatially diversifying selection [31], which is also evident in the present work. Since BayeScan has been suggested to have the lowest error in many contexts [e.g. 63], we only reported results from BayeScan analyses. Despite the many caveats in inferring selection from outlier analyses [see 31, 33], the putatively adaptive SNPs were attempted to be verified through assigning function to them. Along this line it has been suggested to combine DNA, RNA and functional methodologies in field experiments to reveal the genes and mechanisms shaping adaptation in the wild [64]. Factors like temporal dynamics and location of the outlier SNPs in the chromosome can also challenge the inference of selection [65]. Furthermore, the observed pattern using the ‘outlier’ SNPs may simply reflect genetic incompatibilities between the *S*. *mentella* ecotypes [66].

### Extent of hybridization

Compared to our previous work [13], no substantial evidence of hybridization was observed in the present investigation (S7 File). Although statistically significant introgression was estimated by Saha et al. [12] using microsatellites, the extent of hybridization was low, explaining the maintenance of distinct gene pools despite introgression. When *q* values for the ‘shallow’ and ‘deep’ ecotypes were compared, SNP data clearly supported fewer intermediate genotypes (S7 File) than that by microsatellite DNA. The reason for finding lower number of hybrids in the present work may be that STRUCTURE, using a large number of markers and treating them as ‘independent’ (which may not be true), is biased towards assigning fish into ‘purebreds’. This may also partly explain why STRUCTURE failed to identify the ‘slope’ ecotype. Furthermore, we did not include samples from other *Sebastes* species in the present work, rendering interspecific hybrids potentially undetected. The number of hybrids between the ‘shallow’ and the ‘deep’ ecotypes in this study was also lower than in our previous study. When the estimated genetic differentiation is low, as it was in the microsatellite study, STRUCTURE has a tendency to classify ‘pure’ individuals as ‘hybrids’ [55].

### Evolutionary scenario in *S*. *mentella complex*

Our DIYABC computation suggested a concurrent split among the three ecotypes of *S*. *mentella* (Fig 5), although microsatellite DNA [12] indicated a first split between the ‘shallow’ and ‘deep’ ecotypes followed by the split between the ‘shallow’ and ‘slope’ ecotypes. Both Johansen [13] and Daníelsdóttir *et al*. [18] analyzing allozymes suggested a closer connectivity between the ‘slope’ and ‘deep’ ecotypes. Allozymes have fewer loci with lower heterozygosity and subject to selection, lending low power to estimate phylogenetic relationships between the ecotypes. It is also possible that the discrepancy is linked to marker type, with different modes of inheritance, function, and statistical properties [13]. Most importantly, a major reason for the observed difference between SNP and microsatellite results is that the present SNP analysis included all three ecotypes of *S*. *mentella* concurrently using DIYABC method, whereas only two ecotypes of the species were analysed concurrently using the isolation-with-migration method [IM; 67] in our previous microsatellite work. Unlike the IM method, DIYABC assumes no gene flow between the populations after they have split.

For the first time, we provide a complete phylogenetic scenario including all *S*. *mentella* ecotypes (Fig 5). Although Shum et al [17] did not include the ‘slope’ ecotype in their phylogenetic analysis, a similar scenario was supported by their data for the ‘deep’ and ‘shallow’ ecotypes. Our estimates of time since divergence were larger than in Shum et al [16]; however, estimates are comparable with overlapping confidence intervals.

### SNP as tool for population assignment

The present study provides improved tools for investigating genetic population structure in *S*. *mentella*. The 97 outlier loci identified are possibly linked to selection and assigned individuals to the same respective ecotypes and populations as the neutral panel with 1,943 loci. However, only 21 discriminatory SNP loci (16 outliers and 5 neutral SNPs) could assign over 90% of individuals to ecotype and even to population of origin. In Pacific salmon, where population assignment is extensively used in management, a 90% precision in population assignment is considered as the ‘gold standard’ [68]. Our SNP panel with 21 loci will be highly effective at assigning individuals to their population of origin [69], particularly in areas with high levels of mixing such as in Greenlandic waters. The sequence information of these 21 SNPs is available (S9 File), which can easily be used to develop a SNP assay for rapid identification of catch composition in any mixed *S*. *mentella* fishery or even to design a handled device for on-sight discrimination of *S*. *mentella* ecotypes and populations [e.g. 70].

## Supporting information

S1 File Description of the DIYABC analyses and results. (PDF)

S2 File Estimates of minor allele frequency and Hardy-Weinberg proportions of the SNP loci: 4,373 SNPs were selected for 252 fish. (ZIP)

S3 File Descriptive statistics of the SNP loci. Total 4,277 SNPs were found suitable for the downstream analyses. For 1 SNP per RAD tag, 1,943 neutral and 97 outlier loci were selected in the final panels.
(ZIP)

S4 File Results from the analysis of sequence alignment to *Sebastes* genomes. (XLSX)

S5 File Results from the BLAST analyses of outlier SNPs are presented. (XLSX)

S6 File Results from the STRUCTURE analysis. (XLSX)

S7 File Results from the hybrid analyses. (XLSX)

S8 File Accuracy in the population assignment of fish using 21 discriminatory SNPs derived from the *randomForest* analysis. In this figure, each circle represents a fish individual.
(PDF)

S9 File Sequence information for 21 discriminatory SNPs. (XLSX)
