# Supplementary material for: Hierarchical genetic structure in an evolving species complex: Insights from genome wide ddRAD data in Sebastes mentella
Source: PLoS One. 2021 May 27;16(5):e0251976. doi: 10.1371/journal.pone.0251976 (PMC8158871; doi:10.1371/journal.pone.0251976)
Supplement: S1 File — (PDF) [file pone.0251976.s001.pdf]

DIYABC analyses:

In stage/run 1, total 4 populations of *S. mentella* were included and 6 scenarios similar to those of Shum *et al*. (2015) were compared (see figure 1 below). Like Shum *et al*. (2015), we analysed the 'deep' populations from the Faroe Islands and Irminger Sea. These authors analysed the 'shallow' population from the Faroe Islands waters, but we included the 'shallow' population from Norwegian waters (which is not differentiated from the 'shallow' population of the Faroe Islands waters). Unlike Shum *et al*. (2015), we had no 'shallow' sample from the Irminger Sea and we added the 'shallow' fish from nearby Greenland waters instead. Here, scenario 1 proposes original split between the 'shallow' and 'deep' groups, followed by split between the Irminger and Faroe seas within the 'deep' group and between Greenlandic and Norwegian waters within the 'shallow' group. Scenario 2 suggests independent origin of the 'deep' groups from their 'shallow' ancestors in the Norwegian and Greenlandic waters. Unlike scenario 2, scenario 3 has an independent origin of the 'shallow' groups from their 'deep' group ancestors. In scenario 4, 5 and 6, a step-wise divergence was proposed at t1, t2 and t3.

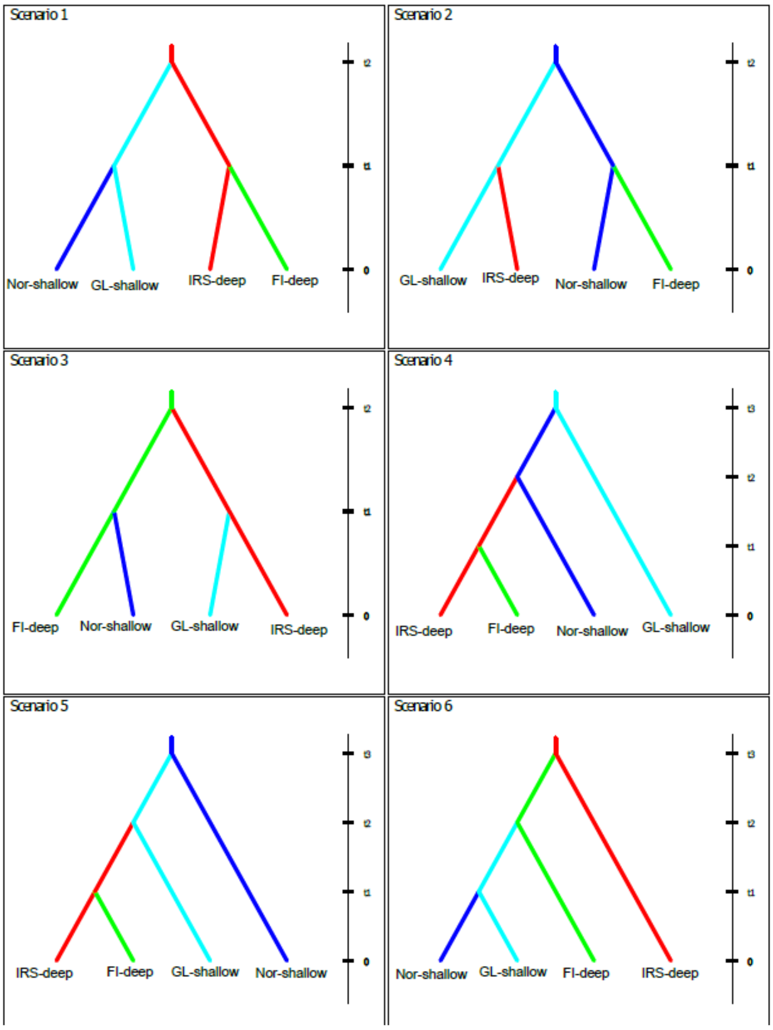

Figure 1: The proposed 6 different scenarios for 4 populations at DIYABC run 1. Here, t3≥t2≥t1.

In stage/run 2, total 5 populations were included (figure 2). Since scenario 1 was supported in run 1 (see result), we simply added the 'slope' group to the scenario 1 of the run 1 and assessed 3 different scenarios in run 2. Here, scenario 1 proposes first split between the 'deep' and 'shallow' groups followed by the second split between the 'shallow' and 'slope' groups. In scenario 2, the second split occurs between the 'deep' and 'slope' groups. Scenario 3 suggests a concurrent split among the 'shallow', 'deep' and 'slope' groups.

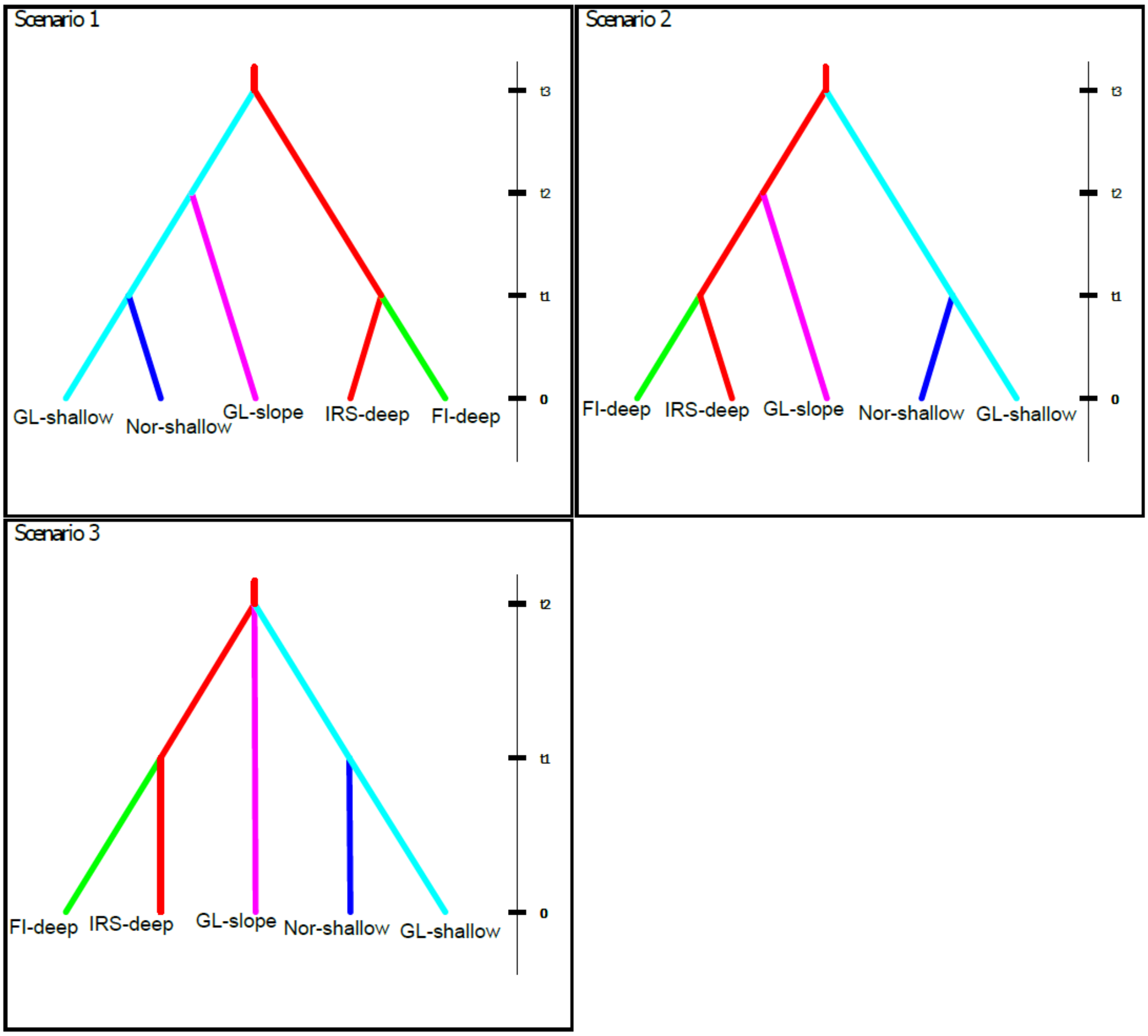

Figure 2: The proposed 3 different scenarios for 5 populations at DIYABC run 2. Here, $t3 \geq t2 \geq t1$.

Other settings:

**Type of statistics**	**Parameters**	**Statistics**	**Number of statistics**
One sample summary statistics	Genic diversities	Mean and variance of non-zero values	8 (4 means and 4 variances for 4 populations)
Two samples summary statistics	F_{ST} distances	Mean and variance of non-zero values	12 (6 means and 6 variances for 6 possible population pairs)
	Nei's distances	Mean and variance of non-zero values	12 (6 means and 6 variances for 6 possible population pairs)

Confidence in scenario choice and model checking

We assessed confidence in scenario choice by evaluating Type I and Type II error rates, following the method described in Cornuet et al. (2010). One thousand test data sets were simulated using each of scenarios. The posterior probability of the competing scenarios was estimated for each of the pseudo-observed data sets. Type I error was estimated by counting the proportion of data sets simulated under the best scenario but resulted in highest posterior probability for other scenarios. Type II error was estimated by the proportion of data sets that resulted in highest posterior probability of the best scenario, although simulated with other scenarios.

For model checking, 1000 data sets were simulated (for each scenario) by drawing with replacement parameter values among the data sets used to compute the posterior distribution of the parameters. The similarity between simulated and real data was estimated using summary statistics differing from the summary statistics used to conduct model choice as suggested by Cornuet et al., (2010). For each summary statistics, the discrepancy between simulated and observed data was assessed.

We assessed precision of parameter estimation by computing the relative median of the absolute error on 500 pseudo-observed data sets simulated with the best scenario. Relative median of the absolute error is the 50% quantile (over the 500 pseudo-observed data sets) of the absolute value of the difference between the median value of the posterior distribution sample (in each data set) and the true value, divided by the true value (Cornuet et al., 2010).

Results from the analyses are presented below

Of the three scenarios studied in the final round analysis, scenario-3 had the highest posterior probability: 78.6% (95%CI: 43%-100%) and 72.5% (95%CI: 45%-100%) using direct and logistics approaches, respectively. Scenario-1 had second highest probability of 12.8% (95% CI:0%-42%, direct method). In round 1 analysis, scenario 1 had the highest posterior probability of 97.2% (95%CI: 93%-100%) using the logistic approach.

The type I error rate using the logistic method in round 1 and 2 were 0.07 (0.03 when only scenario 6 was excluded) and 0.03, respectively. Type II error rates were 0.038 and 0.018 using the logistic method in round 1 and round 2 analyses, respectively.

Model adequacy was assessed for the scenarios by measuring the similarity between the real data set and data sets simulated with each considered scenario under the posterior distribution of parameter values. Similarity was assessed using all available test summary statistics. For the best scenarios less number of observed summary statistics (than those from the other scenarios) deviated significantly from its simulated distribution.

Parameter estimates gave reasonable values which should be reliable because of small relative median of the absolute errors ranging from 0.127 to 0.178 (round 2 analysis). In round 1 analysis, relative median of the absolute errors ranged from 0.27 to 0.44.
