# Supplementary material for: Hierarchical genetic structure in an evolving species complex: Insights from genome wide ddRAD data in Sebastes mentella
Source: PLoS One. 2021 May 27;16(5):e0251976. doi: 10.1371/journal.pone.0251976 (PMC8158871; doi:10.1371/journal.pone.0251976)
Supplement: S8 File — In this figure, each circle represents a fish individual. (PDF) [file pone.0251976.s008.pdf]

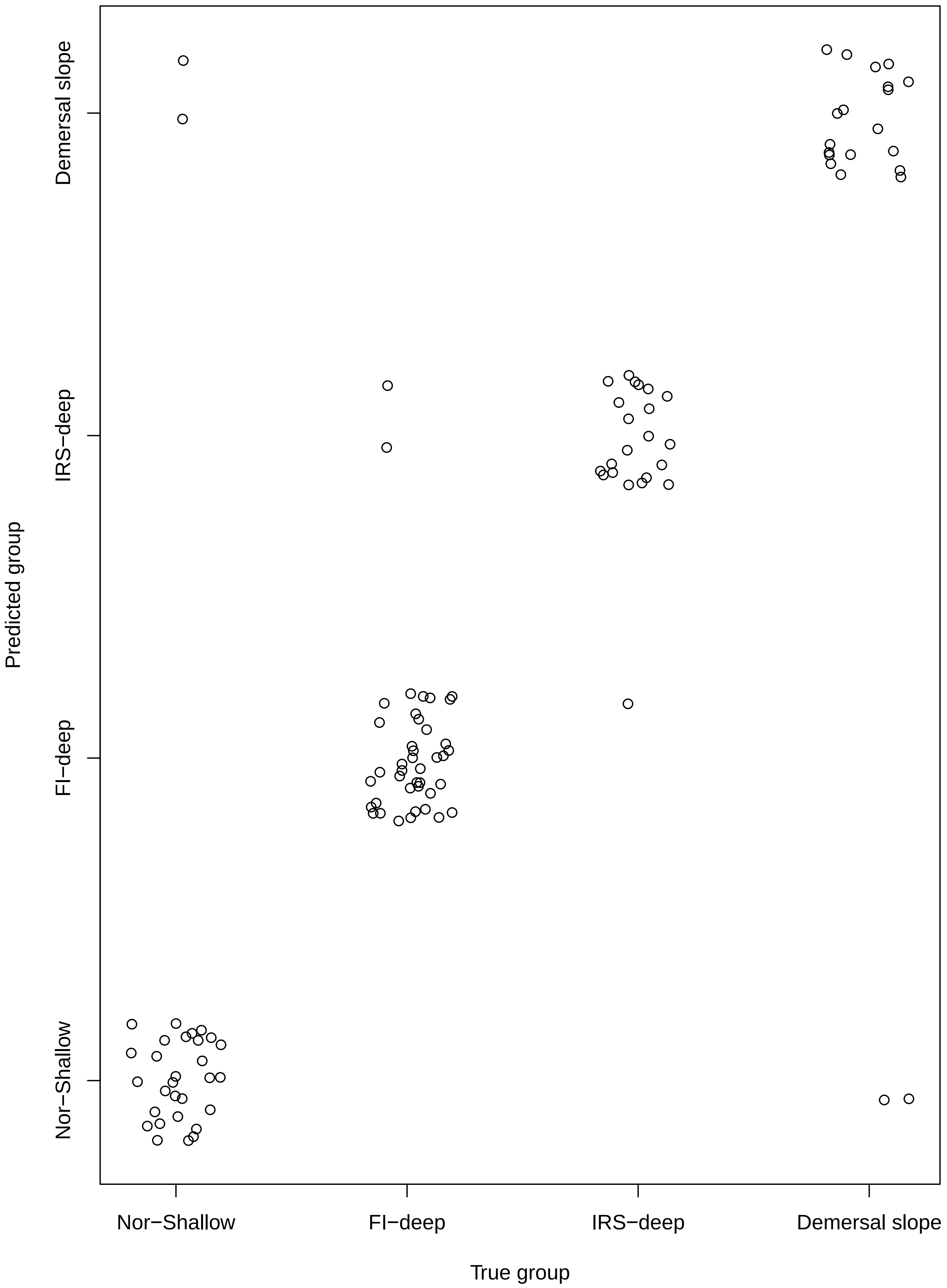
Demersal slope
IRS−deep
FI−deep
Nor−Shallow
Predicted group
Nor−Shallow
FI−deep
IRS−deep
Demersal slope
True group
